# Changes in the CALLY Index After Sleeve Gastrectomy and Their Association with Weight Loss and Metabolic Parameters in Women

**DOI:** 10.3390/jcm15197552

**Published:** 2026-09-29

**Authors:** Süleyman Baş, Gizem Nur Sözen, İlker Kızmaz, Sangar Abdullah, Betül Danapınar, Mustafa Can Şenoymak, Murat Yeniçeri, Kadem Arslan, Alpaslan Tanoğlu

**Affiliations:** 1 Department of Internal Medicine, University of Health Sciences, Sancaktepe Sehit Prof. Dr. Ilhan Varank Training and Research Hospital, 34785 Istanbul, Turkey; 2 Department of Internal Medicine, Hakkari Şemdinli State Hospital, 30800 Hakkari, Turkey; gizemnsozen@gmail.com; 3 Department of Internal Medicine, Mersin Mut State Hospital, 33600 Mersin, Turkey; 4 Department of General Surgery, University of Health Sciences, Sancaktepe Sehit Prof. Dr. Ilhan Varank Training and Research Hospital, 34785 Istanbul, Turkey; 5 Department of Internal Medicine, Karabük University Education and Research Hospital, 78200 Karabük, Turkey; 6 Department of Endocrinology and Metabolism, Hitit University, Çorum Erol Olçok Training and Research Hospital, 19040 Çorum, Turkey; senoymak@gmail.com; 7 Department of Rheumatology, Karaman Training and Research Hospital, 70200 Karaman, Turkey; 8 Division of Gastroenterology, Department of Internal Medicine, Bahçeşehir University, 34732 Istanbul, Turkey

**Keywords:** CALLY index, sleeve gastrectomy, obesity, weight loss, inflammation, insulin resistance

## Abstract

**Background:** The C-reactive protein–albumin–lymphocyte (CALLY) index integrates systemic inflammation, nutritional status, and immune function. This study evaluated changes in the CALLY index after sleeve gastrectomy and their associations with weight loss and cardiometabolic changes in women. **Methods:** This retrospective, observational, single-center study included 72 women who underwent sleeve gastrectomy and had complete preoperative and 12-month postoperative data. The CALLY index was calculated preoperatively and at 12 months. ΔCALLY was defined as the 12-month value minus the preoperative value. Weight-loss outcomes were assessed using total weight loss (TWL) and excess weight loss (EWL). Associations were evaluated using Spearman’s rank correlation analysis and multivariable linear regression. **Results:** The CALLY index increased from 1.70 [0.87–2.56] preoperatively to 6.94 [2.72–15.54] at 12 months (*p* < 0.001). Mean TWL and EWL were 31.43 ± 7.83% and 73.30 ± 20.12%, respectively. ΔCALLY correlated positively with TWL (r = 0.303, *p* = 0.010) and EWL (r = 0.312, *p* = 0.008) and negatively with ΔBMI (r = −0.250, *p* = 0.035), Δfasting insulin (r = −0.234, *p* = 0.046), ΔHOMA-IR (r = −0.243, *p* = 0.040), and Δtriglycerides (r = −0.254, *p* = 0.031). After adjustment for age, preoperative BMI, and diabetes mellitus, ΔCALLY remained positively associated with TWL (B = 0.342, 95% CI 0.172–0.513; *p* < 0.001). **Conclusions:** The CALLY index increased significantly during the first year after sleeve gastrectomy in women. Changes in the CALLY index were associated with weight loss and changes in selected metabolic parameters. ΔCALLY may reflect inflammatory and immunonutritional changes accompanying postoperative weight loss.

## 1. Introduction

Obesity is a chronic and multifactorial disease with a steadily increasing prevalence worldwide and is closely associated with cardiovascular diseases, type 2 diabetes mellitus, dyslipidemia, and other metabolic disorders [1]. Excess adiposity, not limited merely to an increase in energy storage, leads to complex pathophysiological changes characterized by low-grade chronic systemic inflammation, insulin resistance, and various metabolic disorders. Bariatric and metabolic surgery provides not only evident and sustainable weight loss for individuals with severe obesity but also significant improvements in glycemic control, insulin resistance, lipid metabolism, and obesity-related comorbidities. Sleeve gastrectomy is one of the most widely performed bariatric surgery methods due to its effective weight loss and metabolic outcomes [2]. Nevertheless, the evaluation of markers reflecting the inflammatory, immunological, and nutritional changes accompanying postoperative weight loss remains clinically and scientifically relevant.

The C-reactive protein–albumin–lymphocyte (CALLY) index is an immunonutritional biomarker bringing C-reactive protein (CRP), reflecting systemic inflammation; serum albumin, reflecting nutritional status; and lymphocyte count, representing immune function, together into a single composite index [3,4]. Each component of the CALLY index has clinical relevance regarding obesity and bariatric surgery. CRP levels may be elevated in obesity-associated chronic inflammation, whereas the inflammatory burden may decrease following bariatric surgery. Associations between CRP levels and clinical outcomes after bariatric surgery have been reported, while serum albumin has been shown to be associated with nutritional status and postoperative outcomes in patients undergoing bariatric procedures [5,6]. Therefore, the CALLY index, simultaneously reflecting inflammation, nutritional status, and immune response, may be a potentially useful composite marker for evaluating systemic changes following bariatric surgery.

The CALLY index was initially investigated as a prognostic biomarker in malignancies and found to be associated with clinical outcomes in hepatocellular carcinoma, colorectal cancer, and other malignancies [3,4,7]. Recently, the potential role of the CALLY index in the field of bariatric surgery has also begun to be investigated. Radkhah et al. evaluated the relationship between several hematological and immunonutritional indices, including CALLY, and changes in body composition at 3 and 6 months after bariatric surgery in 240 patients [8]. However, evidence regarding the longitudinal behavior of the CALLY index after bariatric surgery remains limited, particularly beyond the early postoperative period. Whether within-patient changes in CALLY over a longer follow-up period are associated with standard bariatric weight-loss measures and concomitant metabolic changes has not been sufficiently characterized.

Therefore, the aim of this study was to evaluate the within-patient change in the CALLY index from the preoperative period to 12 months after sleeve gastrectomy in women and to investigate the associations of this change with standard bariatric weight-loss measures, including TWL and EWL, as well as with changes in glycemic parameters, insulin resistance, and lipid parameters.

## 2. Materials and Methods

### 2.1. Study Design and Population

This retrospective, observational, single-center study included patients who underwent sleeve gastrectomy and were followed at the Obesity Center of Sancaktepe Şehit Prof. Dr. İlhan Varank Training and Research Hospital between 1 April 2023 and 31 December 2024. Patients aged 18–65 years with available preoperative and 12-month postoperative anthropometric and laboratory data required for the calculation of the C-reactive protein–albumin–lymphocyte (CALLY) index were eligible for inclusion. A total of 132 patients were initially screened. Of these, 44 patients were excluded because of incomplete data required for the longitudinal assessment of the CALLY index, leaving 88 patients with complete data. Among these patients, 16 were male, and 72 were female. Because the number of eligible male patients was substantially lower than that of female patients, resulting in a markedly imbalanced sex distribution, the decision was made to restrict the study population to women before the statistical analyses were performed. Consequently, the final study population consisted of 72 women.

Patients with incomplete clinical, anthropometric, or laboratory data at either the preoperative or 12-month postoperative assessment were excluded. Patients with active infection or acute inflammatory conditions at the time of laboratory assessment, active malignancy, chronic inflammatory or autoimmune diseases, hematological disorders affecting lymphocyte counts, advanced liver disease, nephrotic syndrome, or other clinical conditions associated with marked hypoalbuminemia were also excluded. Medication use was assessed using the records of the hospital’s Obesity Center. Patients receiving systemic immunosuppressive therapy or long-term systemic corticosteroid treatment during the preoperative assessment or postoperative follow-up period were excluded because of the potential effects of these treatments on inflammatory and immune parameters. Only patients who completed the 12-month postoperative follow-up were included in the final analysis.

Demographic, clinical, anthropometric, and laboratory data were obtained from the hospital electronic medical records and the records of the hospital’s Obesity Center. Diabetes mellitus, hypertension, and dyslipidemia were identified based on previously documented diagnoses and/or ongoing disease-specific medication use recorded in these sources. Patients without a previously established diagnosis who met the relevant clinical or laboratory diagnostic criteria during the preoperative evaluation were also classified as having the respective comorbidity. Diabetes mellitus was defined as fasting plasma glucose ≥ 126 mg/dL and/or HbA1c ≥ 6.5%. Hypertension was defined as blood pressure ≥ 140/90 mmHg on repeated measurements during the preoperative clinical assessment. Dyslipidemia was defined by ongoing lipid-lowering therapy and/or the presence of an abnormal lipid profile, including total cholesterol ≥ 200 mg/dL, LDL cholesterol ≥ 130 mg/dL, triglycerides ≥ 150 mg/dL, or HDL cholesterol < 50 mg/dL. The patient-selection process is summarized in Figure 1.

### 2.2. Ethical Approval

The study was conducted in accordance with the principles of the Declaration of Helsinki and was approved by the Local Ethics Committee of Sancaktepe Şehit Prof. Dr. İlhan Varank Training and Research Hospital (Decision No: 2026/201; Date: 8 April 2026).

### 2.3. Surgical Procedure

All sleeve gastrectomy procedures were performed laparoscopically by the bariatric surgery team of the Department of General Surgery using a standardized surgical technique. Dissection of the greater curvature was initiated 4–6 cm proximal to the pylorus and continued proximally with division of the short gastric vessels and complete mobilization of the gastric fundus. Gastric transection was performed using a linear stapler over a 38-French bougie positioned along the lesser curvature, beginning 4–6 cm from the pylorus and extending proximally to approximately 0.5–1 cm lateral to the gastroesophageal junction, with complete resection of the fundus. No buttressing material was used, and the staple line was not oversewn or imbricated. Omentopexy of the staple line was performed in all patients using a 3-0 barbed suture. An intraoperative methylene blue leak test was performed in all patients. The same standardized surgical technique was applied throughout the study cohort.

### 2.4. Postoperative Follow-Up and Nutritional Management

On the first postoperative morning, a methylene blue leak test was repeated, and oral fluid intake was initiated when the test was negative. Patients were subsequently followed at the hospital’s Obesity Center, with routine postoperative visits scheduled at 1, 3, 6, and 12 months. Throughout postoperative follow-up, patients were evaluated by the same dietitian team and received dietary counseling as part of the center’s routine follow-up program. Protein supplementation was initiated on postoperative day 3 and continued for the first 3 postoperative months as part of routine nutritional management. Clinical and anthropometric assessments were performed during follow-up visits, together with laboratory evaluations as clinically indicated within the center’s follow-up program. For the present study, the 12-month postoperative anthropometric and laboratory measurements were used for the longitudinal analyses. Postoperative complications and related reinterventions were recorded from the medical records.

### 2.5. Clinical and Anthropometric Assessment

Demographic and clinical data included age, smoking status, and the presence of diabetes mellitus, hypertension, and dyslipidemia. Anthropometric measurements included height and body weight, and body mass index (BMI) was calculated as body weight in kilograms divided by the square of height in meters (kg/m^2^). Preoperative and 12-month postoperative body weight and BMI values were recorded. Systolic and diastolic blood pressure measurements obtained at the corresponding assessments were also recorded.

Postoperative weight-loss outcomes were evaluated using percentage total weight loss (TWL) and percentage excess weight loss (EWL). TWL was calculated as [(preoperative weight − 12-month weight)/preoperative weight] × 100. Ideal body weight was calculated as 25 × height^2^ (m^2^), corresponding to a BMI of 25 kg/m^2^. EWL was subsequently calculated as [(preoperative weight − 12-month weight)/(preoperative weight − ideal body weight)] × 100.

### 2.6. Laboratory Measurements

Laboratory parameters obtained during the preoperative assessment and at the 12-month postoperative follow-up were recorded. Hematological parameters included hemoglobin, total leukocyte count, absolute neutrophil count, absolute lymphocyte count, absolute monocyte count, and platelet count. Inflammatory and nutritional parameters included CRP and serum albumin. The lower detection limit of the CRP assay was 0.10 mg/L. All CRP measurements included in the study were above this limit; therefore, no substitution or imputation for values below the detection limit was required. Glycemic and insulin resistance parameters included fasting plasma glucose, fasting insulin, HbA1c, and the Homeostatic Model Assessment of Insulin Resistance (HOMA-IR). Lipid parameters included total cholesterol, LDL cholesterol, HDL cholesterol, and triglycerides. Liver and renal parameters included alanine aminotransferase (ALT), aspartate aminotransferase (AST), gamma-glutamyl transferase (GGT), and serum creatinine.

HOMA-IR was calculated using fasting glucose and insulin concentrations according to the following formula: HOMA-IR = [fasting insulin (µIU/mL) × fasting glucose (mg/dL)]/405.

### 2.7. Calculation of the CALLY Index

The C-reactive protein–albumin–lymphocyte (CALLY) index was calculated using serum albumin, absolute lymphocyte count, and C-reactive protein (CRP) levels. According to the previously described formula, the CALLY index was calculated as [albumin (g/dL) × absolute lymphocyte count (/µL)]/[CRP (mg/dL) × 10^4^] [4]. In the present study, absolute lymphocyte count and CRP were recorded as ×10^9^/L and mg/L, respectively; therefore, after unit conversion, the mathematically equivalent formula, CALLY index = albumin (g/dL) × absolute lymphocyte count (×10^9^/L)/CRP (mg/L), was used. The CALLY index was calculated separately using preoperative and 12-month postoperative laboratory values. ΔCALLY was defined as the 12-month postoperative CALLY index minus the preoperative CALLY index.

### 2.8. Statistical Analysis

Statistical analyses were performed using IBM SPSS Statistics for Windows, Version 25.0 (IBM Corp., Armonk, NY, USA). The distribution of continuous variables was assessed using the Kolmogorov–Smirnov and Shapiro–Wilk tests. Normally distributed continuous variables are presented as mean ± standard deviation (SD), whereas non-normally distributed variables are expressed as median [interquartile range (IQR)]. Categorical variables are presented as numbers and percentages [*n* (%)]. Preoperative and 12-month postoperative measurements were compared using the paired-samples *t*-test for normally distributed continuous variables and the Wilcoxon signed-rank test for non-normally distributed variables.

Changes in longitudinal variables (Δ) were calculated as the 12-month postoperative value minus the corresponding preoperative value. Associations between ΔCALLY and weight-loss measures (TWL, EWL, and ΔBMI), as well as changes in glycemic, insulin resistance, and lipid parameters, were evaluated using Spearman’s rank correlation analysis. In addition, exploratory Spearman correlation analyses were performed to evaluate the associations between changes in the individual components of the CALLY index (ΔCRP, Δalbumin, and Δlymphocyte count) and TWL. Given the exploratory nature of these correlation analyses, no adjustment for multiple comparisons was applied; therefore, the resulting *p* values should be interpreted as exploratory.

Univariate linear regression analyses were performed to evaluate factors associated with TWL at 12 months. ΔCALLY, preoperative CALLY, age, preoperative BMI, diabetes mellitus, and preoperative HOMA-IR were evaluated individually. A multivariable linear regression model was subsequently constructed with TWL (%) as the dependent variable. ΔCALLY was included as the primary variable of interest, while age, preoperative BMI, and diabetes mellitus were included a priori as clinically relevant potential confounders, irrespective of their statistical significance in univariate analyses. Preoperative HOMA-IR was evaluated only in the univariate analysis and was not included in the primary multivariable model. As a sensitivity analysis, preoperative CALLY was additionally included in the multivariable model to assess whether the association between ΔCALLY and TWL remained after accounting for preoperative CALLY.

Regression results are reported as unstandardized regression coefficients (B) with 95% confidence intervals (CIs). Multicollinearity was assessed using tolerance and variance inflation factor (VIF) values. Model assumptions were evaluated by examining standardized residuals, residual histograms, normal P–P plots, and plots of standardized residuals against standardized predicted values. Potential influential observations were assessed using Cook’s distance. All statistical tests were two-sided, and a *p* value < 0.05 was considered statistically significant.

## 3. Results

### 3.1. Demographic, Clinical, and Anthropometric Characteristics

A total of 72 women who underwent sleeve gastrectomy were included in the study. The mean age of the study population was 38.33 ± 9.53 years, and the mean height was 159.01 ± 6.98 cm. Hypertension, diabetes mellitus, and dyslipidemia were present in 20.8%, 26.4%, and 13.9% of patients, respectively, while 41.7% were current smokers.

Body weight decreased significantly from 112.73 ± 14.18 kg preoperatively to 77.21 ± 12.65 kg at 12 months (*p* < 0.001). Similarly, median BMI decreased from 43.45 [41.16–46.73] kg/m^2^ to 30.20 [26.92–33.66] kg/m^2^ (*p* < 0.001). At 12 months, mean TWL and EWL were 31.43 ± 7.83% and 73.30 ± 20.12%, respectively. Systolic blood pressure decreased significantly from 129.69 ± 15.97 mmHg to 115.90 ± 10.01 mmHg (*p* < 0.001), while diastolic blood pressure decreased from 83.03 ± 11.72 mmHg to 76.29 ± 8.46 mmHg (*p* = 0.001). The demographic, clinical, and anthropometric characteristics of the study population are presented in Table 1.

Regarding postoperative complications, one patient (1.4%) required surgical reintervention on postoperative day 1 because of a thermal injury to the gastric fundus adjacent to the staple line. The injury was successfully managed by wedge resection.

### 3.2. Changes in Laboratory Parameters and the CALLY Index

Significant changes were observed in several hematological, inflammatory, glycemic, metabolic, and biochemical parameters following sleeve gastrectomy (Table 2). Leukocyte, neutrophil, monocyte, and platelet counts decreased significantly at 12 months (all *p* < 0.001), whereas the change in lymphocyte count was not statistically significant (*p* = 0.214). A modest but significant decrease in hemoglobin was also observed (*p* = 0.015).

A marked reduction in systemic inflammation was evident, with median CRP decreasing from 6.22 [4.30–11.28] mg/L preoperatively to 1.43 [0.63–3.80] mg/L at 12 months (*p* < 0.001). In contrast, serum albumin levels remained relatively stable (*p* = 0.077). Notably, the median CALLY index increased substantially from 1.70 [0.87–2.56] preoperatively to 6.94 [2.72–15.54] at 12 months (*p* < 0.001). The 12-month postoperative CALLY index ranged from 0.210 to 52.601, while ΔCALLY ranged from −7.945 to 49.059.

Significant improvements were also observed in glycemic and insulin resistance parameters. Fasting glucose, fasting insulin, HOMA-IR, and HbA1c all decreased significantly at 12 months (all *p* < 0.001). Regarding the lipid profile, HDL cholesterol increased significantly, while triglyceride levels decreased (both *p* < 0.001); no significant changes were observed in LDL or total cholesterol. ALT, AST, and GGT levels also decreased significantly (all *p* < 0.001), whereas serum creatinine remained unchanged.

### 3.3. Correlations Between Changes in CALLY Index and Weight Loss and Metabolic Parameters

The change in the CALLY index (ΔCALLY) was significantly correlated with postoperative weight-loss parameters (Table 3). ΔCALLY showed positive correlations with TWL (r = 0.303, *p* = 0.010) and EWL (r = 0.312, *p* = 0.008) and a negative correlation with ΔBMI (r = −0.250, *p* = 0.035).

ΔCALLY was also significantly correlated with changes in selected metabolic parameters. Negative correlations were observed with changes in fasting insulin (r = −0.234, *p* = 0.046), HOMA-IR (r = −0.243, *p* = 0.040), and triglycerides (r = −0.254, *p* = 0.031). Correlations with changes in fasting glucose (r = −0.220, *p* = 0.063), HbA1c (r = −0.131, *p* = 0.332), HDL cholesterol (r = 0.188, *p* = 0.115), LDL cholesterol (r = −0.214, *p* = 0.072), and total cholesterol (r = −0.161, *p* = 0.177) did not reach statistical significance.

In an exploratory analysis of the individual components of the CALLY index, changes in CRP (r = −0.178, *p* = 0.134) and albumin (r = −0.069, *p* = 0.565) were not significantly correlated with TWL. A weak inverse correlation was observed between the change in lymphocyte count and TWL (r = −0.234, *p* = 0.048). Although ΔCALLY was significantly correlated with TWL, the change in CRP alone was not significantly correlated with TWL.

### 3.4. Factors Associated with Total Weight Loss at 12 Months

In univariate linear regression analyses, ΔCALLY was positively associated with TWL at 12 months (B = 0.299, 95% CI 0.130–0.468; *p* = 0.001), whereas preoperative CALLY, age, preoperative BMI, diabetes mellitus, and preoperative HOMA-IR were not significantly associated with TWL (Table 4).

In the multivariable linear regression model adjusted for age, preoperative BMI, and diabetes mellitus, ΔCALLY remained significantly and positively associated with TWL (adjusted B = 0.342, 95% CI 0.172–0.513; *p* < 0.001). Age was also inversely associated with TWL, with increasing age associated with lower TWL (adjusted B = −0.250, 95% CI −0.431 to −0.070; *p* = 0.007). Preoperative BMI and diabetes mellitus were not significantly associated with TWL.

The overall multivariable model was statistically significant (F[4,67] = 5.340, *p* = 0.001), explaining 24.2% of the variance in TWL (R^2^ = 0.242; adjusted R^2^ = 0.196). No evidence of multicollinearity was identified, and residual diagnostics indicated no major violations of the assumptions of linear regression.

In a sensitivity analysis additionally including the preoperative CALLY index, ΔCALLY remained significantly and positively associated with TWL (B = 0.353, 95% CI 0.178–0.529; *p* < 0.001), whereas preoperative CALLY was not significantly associated with TWL (B = −0.245, 95% CI −1.048 to 0.559; *p* = 0.545). The sensitivity model was statistically significant (F[5,66] = 4.306, *p* = 0.002; R^2^ = 0.246; adjusted R^2^ = 0.189), with no evidence of multicollinearity (VIF range, 1.068–1.142).

## 4. Discussion

In this study, the CALLY index increased significantly at 12 months compared with the preoperative period in women who underwent sleeve gastrectomy. Changes in the CALLY index were significantly associated with postoperative weight-loss measures, with ΔCALLY showing positive correlations with total weight loss (TWL) and excess weight loss (EWL) and a negative correlation with ΔBMI. ΔCALLY was also significantly associated with changes in fasting insulin, HOMA-IR, and triglyceride levels. The association between ΔCALLY and TWL remained significant after adjustment for age, preoperative BMI, and diabetes mellitus. These findings suggest that changes in the CALLY index following sleeve gastrectomy are associated with postoperative weight loss and selected metabolic changes and may reflect inflammatory and immunonutritional changes accompanying weight loss.

In our study, the marked increase in the CALLY index at 12 months appeared to occur predominantly in parallel with the substantial reduction in systemic inflammatory burden. Because the CALLY index integrates CRP, albumin, and lymphocyte count into a single composite measure, changes in the index may reflect alterations across inflammatory, nutritional, and immune components [3,4]. CRP levels decreased markedly at 12 months postoperatively, whereas no significant changes were observed in serum albumin or lymphocyte count. At the group level, this pattern suggests that the postoperative increase in the CALLY index was largely driven by the reduction in CRP. However, the exploratory component analysis provided a more nuanced picture at the individual level. Although ΔCALLY was significantly correlated with TWL, the change in CRP alone was not significantly correlated with TWL. Changes in albumin were also not associated with TWL, while the association with change in lymphocyte count was weak and should be interpreted cautiously, given the exploratory nature of these analyses and the absence of adjustment for multiple comparisons. Therefore, the observed association between ΔCALLY and TWL cannot be attributed solely to the relationship between CRP reduction and weight loss, although these findings do not establish that CALLY provides additional or superior information compared with CRP alone.

Our findings are consistent with previous studies demonstrating reductions in systemic inflammation after sleeve gastrectomy. Randell et al. reported that CRP levels decreased significantly following sleeve gastrectomy in 197 patients and that this reduction persisted for up to 24 months; moreover, the change in CRP at 12 months was associated with changes in body weight [9]. Similarly, Lo et al. demonstrated improvements in inflammatory and immune profiles during a 12-month prospective follow-up, accompanied by reductions in CRP, leukocyte, and neutrophil levels [10]. More recent longitudinal data also support that low-grade systemic inflammation decreases significantly within 12 months following bariatric surgery [11]. Taken together, these findings support the marked postoperative reduction in inflammatory burden observed in our cohort, while the clinical significance of the accompanying increase in the CALLY index requires further investigation.

Direct evidence regarding changes in the CALLY index following bariatric surgery remains limited. Radkhah et al. evaluated the relationship between several immunonutritional indices, including CALLY, and changes in body composition at 3 and 6 months after bariatric surgery in 240 patients and reported that the glucose-to-lymphocyte ratio (GLR), prognostic nutritional index (PNI), and hemoglobin–albumin–lymphocyte–platelet (HALP) index, in particular, were associated with early postoperative changes in body composition [8]. In contrast, our study focused on the 12-month within-patient change in the CALLY index (ΔCALLY) and evaluated its associations with TWL and EWL, as well as with changes in glycemic and lipid parameters. Notably, preoperative CALLY was not associated with TWL, whereas the association between ΔCALLY and TWL remained significant in the sensitivity analysis after accounting for preoperative CALLY. This finding suggests that the longitudinal change in the CALLY index, rather than its preoperative value alone, may be more closely associated with postoperative weight loss in our cohort. In this regard, our findings extend the limited literature by providing 12-month longitudinal data on the relationship between postoperative changes in the CALLY index, weight loss, and concomitant metabolic changes.

The association between changes in the CALLY index and postoperative weight loss is one of the notable findings of our study. ΔCALLY was positively correlated with both TWL and EWL and negatively correlated with ΔBMI. These mathematically related measures represent complementary expressions of the same postoperative weight-loss process, and their consistent correlations with ΔCALLY support the overall association between changes in the CALLY index and postoperative weight loss rather than representing independent confirmations. This relationship was further supported by the multivariable analysis, in which ΔCALLY remained positively associated with TWL at 12 months after adjustment for age, preoperative BMI, and diabetes mellitus. Chronic low-grade inflammation accompanying obesity is known to be closely linked to inflammatory and immune processes within adipose tissue [12]. Longitudinal evidence indicates substantial improvements in low-grade systemic inflammation following bariatric surgery. Jensen et al. evaluated a broad panel of inflammatory proteins in patients undergoing bariatric surgery and demonstrated that a significant proportion of inflammatory markers elevated at baseline decreased by the 12th postoperative month [11]. Similarly, 10-year follow-up data from the SLEEVEPASS randomized clinical trial demonstrated sustained reductions in hs-CRP following bariatric surgery and an association between greater TWL and lower hs-CRP levels at 10 years [13]. In this context, the association between ΔCALLY and postoperative weight loss observed in our study may reflect changes in obesity-related inflammatory burden together with changes in immunonutritional status. However, because of the observational nature of the study, the directionality and causality of this association cannot be established. Therefore, ΔCALLY should not be interpreted as a predictor of postoperative weight loss but rather as a potential marker reflecting inflammatory and immunonutritional changes accompanying weight loss.

In our study, the negative correlations of ΔCALLY with changes in fasting insulin, HOMA-IR, and triglyceride levels suggest that changes in the CALLY index may be associated not only with weight loss but also with selected metabolic changes following sleeve gastrectomy. Improvements in insulin sensitivity following sleeve gastrectomy have been linked not only to weight loss but also to reductions in visceral adiposity, attenuation of inflammatory signaling pathways, and hormonal and metabolic adaptations [14]. In a prospective cohort of 199 patients undergoing sleeve gastrectomy, Davoudi et al. reported significant decreases in serum insulin and HOMA-IR, together with improvements in lipid profiles and inflammatory markers, six months after surgery [14]. Similarly, in a more recent bariatric surgery cohort, significant reductions in fasting glucose, HbA1c, triglyceride, and HOMA-IR levels, together with an increase in HDL levels, were observed postoperatively [15]. These findings support the concept that inflammatory and metabolic improvements may occur in parallel following bariatric surgery. In our study, the associations of ΔCALLY with changes in fasting insulin, HOMA-IR, and triglyceride levels suggest that changes in the CALLY index may accompany selected metabolic changes after sleeve gastrectomy. However, these correlation analyses were exploratory and were not adjusted for multiple comparisons; therefore, the observed associations should be interpreted cautiously. Moreover, the absence of significant associations between ΔCALLY and changes in fasting glucose, HbA1c, HDL, LDL, and total cholesterol suggests that CALLY should not be regarded as a general marker that uniformly reflects all cardiometabolic changes.

Postoperative complications are an important aspect of clinical outcomes after bariatric surgery. In the present study, only one patient (1.4%) required early surgical reintervention because of a thermal injury to the gastric fundus adjacent to the staple line, which was successfully managed by wedge resection. Although uncommon, complications such as leaks and fistulas remain clinically important after bariatric surgery and may require complex multidisciplinary management, including endoscopic interventions [16,17]. The low frequency of postoperative complications in our cohort should therefore be considered when interpreting our findings, as the observed changes in the CALLY index primarily reflect the postoperative course of women without major complications. Accordingly, these findings may not be directly generalizable to patients experiencing complicated postoperative courses.

### 4.1. Strengths

Our study has several strengths. First, the within-patient assessment of the CALLY index from the preoperative period to 12 months after sleeve gastrectomy enabled us to evaluate longitudinal changes in the index alongside weight loss and metabolic changes. Rather than evaluating CALLY at a single time point, we focused on its individual change (ΔCALLY) and examined its associations with standard bariatric weight-loss measures, including TWL and EWL, as well as with changes in glycemic control, insulin resistance, and lipid parameters. In addition, exploratory analyses of the individual CALLY components allowed the relationship between ΔCALLY and TWL to be considered in the context of concurrent changes in CRP, albumin, and lymphocyte count. The association between ΔCALLY and TWL was further evaluated in a multivariable model adjusted for clinically relevant potential confounders, including age, preoperative BMI, and diabetes mellitus, and remained significant in a sensitivity analysis additionally accounting for preoperative CALLY. Finally, major clinical conditions and treatments that could substantially affect the components of the CALLY index were addressed in the exclusion criteria, including systemic immunosuppressive therapy and long-term systemic corticosteroid use, thereby limiting the potential influence of these factors on postoperative changes in the index.

### 4.2. Limitations

Several limitations of the study should be acknowledged. First, the single-center design and relatively small sample size limit the generalizability of the findings and restrict the number of potential confounders that can be evaluated, particularly in multivariable analyses. Because the number of eligible male patients was substantially lower than that of female patients, resulting in a markedly imbalanced sex distribution, the study population was restricted to women; therefore, potential sex-related differences could not be assessed, and the findings cannot be directly generalized to men. In addition, the exclusion of patients with incomplete data required for longitudinal CALLY assessment may have introduced attrition and selection bias, as the final analysis was restricted to patients with complete preoperative and 12-month data. Patients who completed the 12-month assessment may have differed systematically from those who did not return for follow-up with respect to clinical characteristics, adherence to postoperative care, or weight-loss outcomes, which may limit the generalizability of the findings. The observational design precludes establishing the directionality or causality of the associations between ΔCALLY and weight loss or metabolic changes. Although major clinical conditions and treatments that could substantially affect the components of the CALLY index were addressed in the exclusion criteria, residual confounding from unmeasured nutritional and inflammatory factors cannot be excluded. Furthermore, longitudinal changes in medications for obesity-related comorbidities were not systematically captured as a predefined study variable. Therefore, the potential effects of changes in antidiabetic, antihypertensive, and lipid-lowering therapies on the observed metabolic changes could not be fully accounted for and may represent an additional source of residual confounding. The low frequency of postoperative complications in our cohort may also limit the generalizability of the findings to patients experiencing complicated postoperative courses. Finally, because the CALLY index and metabolic parameters were assessed only preoperatively and at 12 months postoperatively, the study does not allow detailed evaluation of early postoperative changes or the trajectory of the CALLY index over time. Prospective multicenter studies with larger populations, inclusion of both sexes, and assessments at multiple postoperative time points are needed to validate these findings.

## 5. Conclusions

In this study, the CALLY index increased significantly at 12 months after sleeve gastrectomy compared with the preoperative period in women. Changes in the CALLY index were associated with postoperative weight loss and changes in selected metabolic parameters, including fasting insulin, HOMA-IR, and triglyceride levels. The association between ΔCALLY and TWL remained significant after adjustment for age, preoperative BMI, and diabetes mellitus. These findings suggest that ΔCALLY may reflect inflammatory and immunonutritional changes accompanying postoperative weight loss and selected metabolic changes after sleeve gastrectomy. However, the clinical significance of postoperative changes in the CALLY index remains to be established, and prospective multicenter studies with larger populations, including both sexes, are needed to validate these findings.

## Figures and Tables

**Figure 1:**
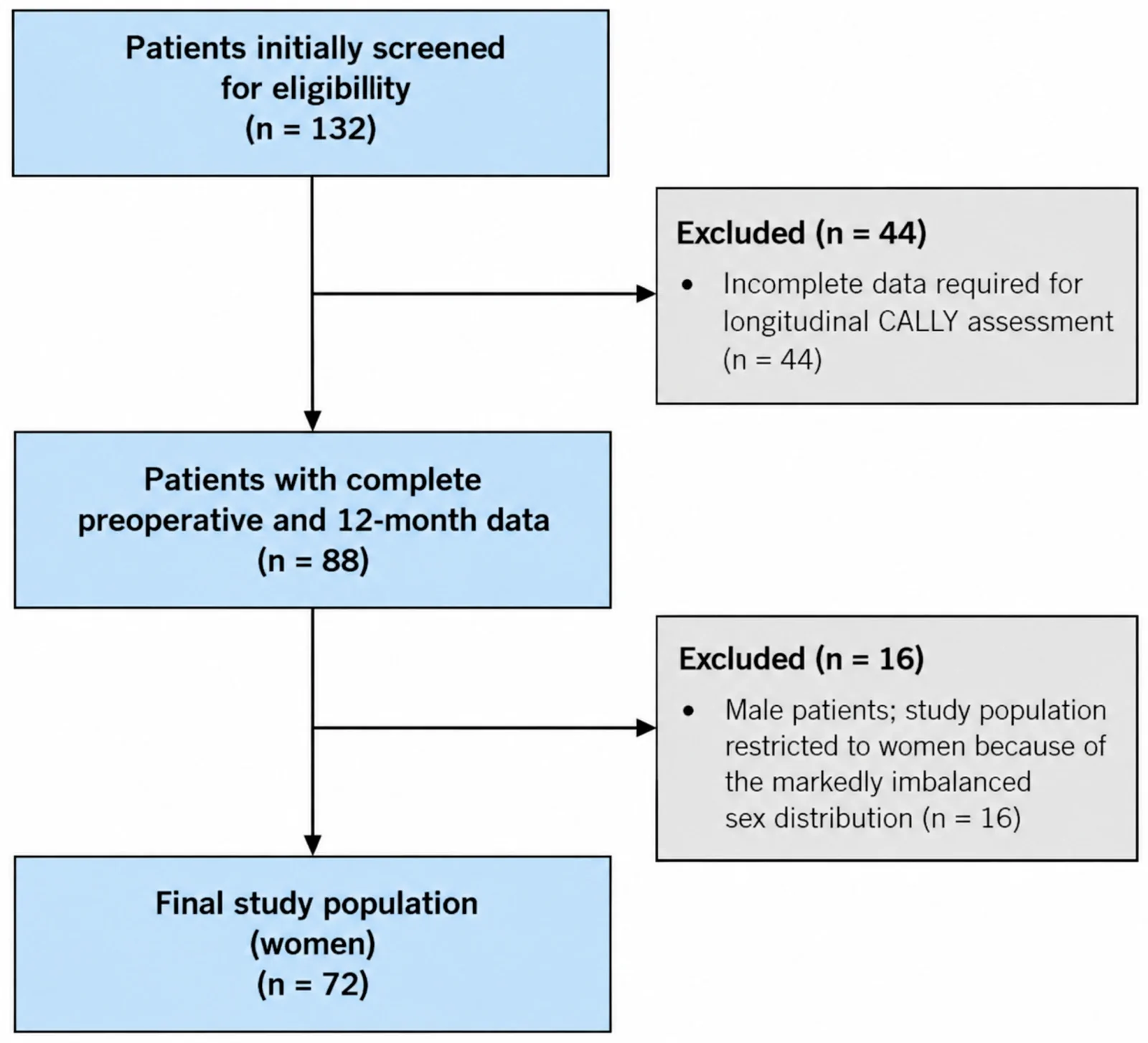
Flowchart of the study population.

**Table 1:** Demographic, clinical, and anthropometric characteristics of the study population.

Variable	Patients (*n* = 72)	*p*
**Age (years)**	38.33 ± 9.53 ^a^	—
**Height (cm)**	159.01 ± 6.98 ^a^	—
**Body weight (kg)**		<0.001 ^c,^*
Preoperative	112.73 ± 14.18 ^a^	
12 months	77.21 ± 12.65 ^a^	
**BMI (kg/m^2^)**		<0.001 ^d,^*
Preoperative	43.45 [41.16–46.73] ^b^	
12 months	30.20 [26.92–33.66] ^b^	
**TWL (%)**	31.43 ± 7.83 ^a^	
**EWL (%)**	73.30 ± 20.12 ^a^	
**Systolic blood pressure (mmHg)**		<0.001 ^c,^*
Preoperative	129.69 ± 15.97 ^a^	
12 months	115.90 ± 10.01 ^a^	
**Diastolic blood pressure (mmHg)**		0.001 ^c,^*
Preoperative	83.03 ± 11.72 ^a^	
12 months	76.29 ± 8.46 ^a^	
**Comorbidities**		
Hypertension	15 (20.8%)	—
Diabetes mellitus	19 (26.4%)	—
Dyslipidemia	10 (13.9%)	—
**Current smoking**	30 (41.7%)	—

^a^: mean ± standard deviation (SD); ^b^: median [interquartile range (IQR)]; ^c^: paired-samples *t*-test; ^d^: Wilcoxon signed-rank test; *: statistically significant (*p* < 0.05); BMI: body mass index; TWL: total weight loss; EWL: excess weight loss.

**Table 2:** Comparison of laboratory parameters and the CALLY index between preoperative and 12-month postoperative assessments.

Parameter	Preoperative	12 Months	*p*
**Hematological parameters**			
Hemoglobin (g/dL)	12.86 ± 1.12 ^a^	12.56 ± 1.27 ^a^	0.015 ^c,^*
Leukocyte count (×10^9^/L)	8.11 ± 1.99 ^a^	6.89 ± 1.92 ^a^	<0.001 ^c,^*
Neutrophil count (×10^9^/L)	5.06 ± 1.52 ^a^	4.03 ± 1.44 ^a^	<0.001 ^c,^*
Lymphocyte count (×10^9^/L)	2.39 [1.95–2.72] ^b^	2.21 [1.80–2.68] ^b^	0.214 ^d^
Monocyte count (×10^9^/L)	0.40 [0.35–0.52] ^b^	0.37 [0.30–0.41] ^b^	<0.001 ^d,^*
Platelet count (×10^9^/L)	294.69 ± 66.96 ^a^	243.43 ± 61.39 ^a^	<0.001 ^c,^*
**Inflammatory and nutritional parameters**			
CRP (mg/L)	6.22 [4.30–11.28] ^b^	1.43 [0.63–3.80] ^b^	<0.001 ^d,^*
Albumin (g/dL)	4.42 ± 0.25 ^a^	4.35 ± 0.33 ^a^	0.077 ^c^
CALLY index	1.70 [0.87–2.56] ^b^	6.94 [2.72–15.54] ^b^	<0.001 ^d,^*
**Glycemic and insulin resistance parameters**			
Fasting glucose (mg/dL)	98 [91.17–109.25] ^b^	83.05 [79.25–90.75] ^b^	<0.001 ^d,^*
Fasting insulin (µIU/mL)	17.30 [10.70–25] ^b^	6.15 [4.42–10.27] ^b^	<0.001 ^d,^*
HOMA-IR	4.32 [2.51–7.73] ^b^	1.27 [0.76–2.04] ^b^	<0.001 ^d,^*
HbA1c (%)	5.80 [5.50–6.10] ^b^	5.31 [5.20–5.60] ^b^	<0.001 ^d,^*
Lipid parameters			
HDL cholesterol (mg/dL)	48.15 [42.62–54] ^b^	57 [49.40–67.80] ^b^	<0.001 ^d,^*
LDL cholesterol (mg/dL)	118.75 ± 36.26 ^a^	116.81 ± 37.07 ^a^	0.508 ^c^
Total cholesterol (mg/dL)	199.15 ± 42.83 ^a^	193.23 ± 40.89 ^a^	0.165 ^c^
Triglyceride (mg/dL)	150.50 ± 65.43 ^a^	90.35 ± 37.87 ^a^	<0.001 ^c,^*
**Liver and renal function parameters**			
ALT (U/L)	18.30 [13.57–23.87] ^b^	11.10 [8.52–15.35] ^b^	<0.001 ^d,^*
AST (U/L)	16.60 [14.50–20.75] ^b^	13.20 [10.70–16.20] ^b^	<0.001 ^d,^*
GGT (U/L)	21.40 [16–31.22] ^b^	9.45 [7.67–15.32] ^b^	<0.001 ^d,^*
Creatinine (mg/dL)	0.68 ± 0.11 ^a^	0.67 ± 0.12 ^a^	0.506 ^c^

^a^: mean ± standard deviation (SD); ^b^: median [interquartile range (IQR)]; ^c^: paired-samples *t*-test; ^d^: Wilcoxon signed-rank test; *: statistically significant (*p* < 0.05).

**Table 3:** Correlations between change in CALLY index and weight loss and metabolic changes at 12 months.

	ΔCALLY	
Variable	r	*p*
**Weight loss and anthropometric parameters**		
TWL (%)	**0.303**	0.010 *
EWL (%)	**0.312**	0.008 *
ΔBMI (kg/m^2^)	**−0.250**	0.035 *
**Glycemic and insulin resistance parameters**		
ΔFasting glucose (mg/dL)	−0.220	0.063
ΔFasting insulin (µIU/mL)	**−0.234**	0.046 *
ΔHOMA-IR	**−0.243**	0.040 *
ΔHbA1c (%)	−0.131	0.332
**Lipid parameters**		
ΔHDL cholesterol (mg/dL)	0.188	0.115
ΔLDL cholesterol (mg/dL)	−0.214	0.072
ΔTotal cholesterol (mg/dL)	−0.161	0.177
ΔTriglyceride (mg/dL)	**−0.254**	0.031 *
**CALLY component changes in relation to TWL**		
ΔCRP vs. TWL	−0.178	0.134
ΔAlbumin vs. TWL	−0.069	0.565
ΔLymphocyte count vs. TWL	**−0.234**	0.048 *

Correlations were assessed using Spearman’s rank correlation analysis. Δ was calculated as the 12-month postoperative value minus the preoperative value. For weight loss, anthropometric, glycemic, insulin resistance, and lipid parameters, correlation coefficients represent their associations with ΔCALLY. For the CALLY component analysis, correlations between changes in CRP, albumin, and lymphocyte count and TWL were evaluated separately. Correlation analyses were considered exploratory, and *p* values were not adjusted for multiple comparisons; therefore, the findings should be interpreted cautiously. *: Statistically significant (*p* < 0.05). TWL: total weight loss; EWL: excess weight loss; BMI: body mass index; HOMA-IR: Homeostatic Model Assessment for Insulin Resistance.

**Table 4:** Univariate, multivariable, and sensitivity analyses of factors associated with total weight loss at 12 months.

Variable	Univariate B (95% CI)	*p*	Adjusted B (95% CI)	*p*	Baseline CALLY-Adjusted Sensitivity Model B (95% CI)	*p*
ΔCALLY index	**0.299** **(0.130 to 0.468)**	0.001 *	**0.342** **(0.172 to 0.513)**	<0.001 *	**0.353** **(0.178 to 0.529)**	<0.001 *
Preoperative CALLY index	0.107 (−0.754 to 0.968)	0.805	—	—	−0.245 (−1.048 to 0.559)	0.545
Age (years)	−0.184 (−0.375 to 0.006)	0.058	**−0.250** **(−0.431 to −0.070)**	0.007 *	**−0.253** **(−0.434 to −0.071)**	0.007 *
Preoperative BMI (kg/m^2^)	−0.020 (−0.482 to 0.442)	0.932	−0.028 (−0.446 to 0.390)	0.893	−0.053 (−0.480 to 0.375)	0.806
Diabetes mellitus	0.845 (−3.720 to 5.409)	0.713	−0.084 (−4.426 to 4.259)	0.969	−0.131 (−4.498 to 4.236)	0.953
Preoperative HOMA-IR	0.050 (−0.192 to 0.293)	0.680	—	—	—	—

B represents the unstandardized regression coefficient. The dependent variable was total weight loss (TWL, %). The main multivariable model included ΔCALLY index, age, preoperative BMI, and diabetes mellitus. The sensitivity model additionally included the preoperative CALLY index. All variables were entered simultaneously. ΔCALLY was calculated as the 12-month postoperative value minus the preoperative value. Diabetes mellitus was coded as 0 = no and 1 = yes. All analyses included 72 patients. The main multivariable model was statistically significant (R^2^ = 0.242, adjusted R^2^ = 0.196; F[4,67] = 5.340, *p* = 0.001), as was the sensitivity model (R^2^ = 0.246, adjusted R^2^ = 0.189; F[5,66] = 4.306, *p* = 0.002). No evidence of multicollinearity was observed in either model (main model VIF range, 1.031–1.139; sensitivity model VIF range, 1.068–1.142). *: Statistically significant (*p* < 0.05). CI: confidence interval; VIF: variance inflation factor; BMI: body mass index; TWL: total weight loss; HOMA-IR: Homeostatic Model Assessment for Insulin Resistance.

## Data Availability

The data are available from the corresponding author upon reasonable request due to ethical and confidentiality restrictions.
